# Construction of a predictive model for type 2 diabetes mellitus with coexisting hypertension: A cross-sectional study

**DOI:** 10.1097/MD.0000000000041047

**Published:** 2025-01-03

**Authors:** Huiling Zhang, Shuang Yu, Zheyuan Xia, Yahui Meng, Dezheng Zhu, Xiang Wang, Hui Shi

**Affiliations:** a School of Nursing, Anhui University of Traditional Chinese Medicine, Hefei, China; b College of Nursing and Allied Health Sciences, St. Paul University Manila, Malate, Metro Manila, Philippines; c The Hospital of Suixi County, Suixi County, Anhui Province, China.

**Keywords:** hypertension, logistic regression, nomogram, predictive model, risk assessment, type 2 diabetes

## Abstract

Type 2 diabetes mellitus (T2DM) and hypertension often coexist, raising the risk of cardiovascular events, renal disease, and mortality. Early identification of high-risk patients with T2DM and concurrent HTN is vital for personalized care. This study aims to construct and validate a predictive model for hypertension in T2DM patients to aid early intervention and tailored treatment. A quantitative observational study using multivariable logistic regression analysis was conducted, with results presented in a nomogram. Data from 423 T2DM patients (206 with hypertension and 217 without) hospitalized at a tertiary hospital in Anhui Province between February 2023 and February 2024 were analyzed. Univariate and multivariate logistic regression identified significant predictors, and model performance was evaluated via ROC curves, AUC values, and the Hosmer–Lemeshow test. Age, alcohol use, diabetic nephropathy, coronary heart disease, cerebral infarction, and body mass index were significant predictors. The model showed good performance with an AUC of 0.72, and the Hosmer–Lemeshow test (*P* = .074) confirmed its fit. The predictive model effectively identifies high-risk T2DM patients for hypertension, aiding early intervention and personalized treatment.

## 1. Introduction

Type 2 diabetes mellitus (T2DM) and hypertension are major global public health issues. These 2 diseases often coexist and interact, increasing the risk of cardiovascular events, kidney disease, and all-cause mortality.^[1]^ Studies have shown that approximately 50% of T2DM patients suffer from hypertension, and the coexistence of these conditions significantly elevates the incidence and mortality rates associated with cardiovascular diseases.^[2]^ Therefore, constructing an effective predictive model for the risk of hypertension in patients with T2DM is particularly important. In managing T2DM and hypertension, traditional treatment approaches primarily focus on controlling blood glucose and blood pressure levels.^[3]^ However, the importance of individualized risk prediction is often overlooked. Early identification of high-risk individuals with coexisting hypertension among T2DM patients can facilitate personalized treatment strategies, optimize resource allocation, and reduce the incidence of long-term complications.

At present, some studies have attempted to construct predictive models for hypertension in T2DM patients, aiming to identify high-risk individuals at an early stage of the disease, allowing for timely intervention. These models are primarily based on common clinical indicators such as age, gender, body mass index (BMI), blood glucose levels, and lipid profiles.^[4]^ By analyzing the association between these indicators and the onset of hypertension, these models provide valuable insights. However, these models still need to improve. Firstly, most predictive models fail to adequately consider the complex interactions between T2DM and hypertension, often relying solely on simple linear regression or logistic regression methods.^[5]^ Additionally, few models incorporate new biomarker data, which may be crucial for the early identification of hypertension.^[6]^ Therefore, this study aims to fill the gaps in previous research by developing a more comprehensive and accurate predictive model for T2DM with coexisting hypertension. By employing this comprehensive and refined risk assessment approach, high-risk individuals can be identified more accurately, allowing for personalized prevention and management strategies and ultimately optimizing overall patient health outcomes.

The aim of our study is to develop a predictive model based on independent risk factors to identify high-risk patients for hypertension among those with T2DM. The goal of this predictive model is to assist clinicians in quickly assessing a patient’s risk of hypertension based on their individual characteristics, thus providing a basis for early intervention and personalized treatment.

## 2. Methods

### 2.1. Study design and participants

A total of 423 cases of T2DM patients, meeting the inclusion and exclusion criteria, were collected from the Endocrinology Department of a Tertiary Hospital in Anhui Province from February 1, 2023, to February 1, 2024. Among these patients, 206 had coexisting hypertension (diabetes with hypertension group), while 217 had normal blood pressure (diabetes group). The study was approved by the hospital’s ethics committee under approval number 2023AH-23. All participants adhered to the principles of informed consent and confidentiality. All data were handled with confidentiality.

### 2.2. Inclusion criteria

All patients must meet the T2DM diagnostic criteria as outlined in the “Chinese Guidelines for the Prevention and Treatment of Type 2 Diabetes.”

Patients must meet the hypertension diagnostic criteria as outlined in the “Chinese Guidelines for the Prevention and Treatment of Hypertension.”

Complete case data must be available; for patients with multiple hospital admissions, clinical data from the first admission were collected.

Patients must have no language communication barriers, demonstrate good compliance, voluntarily participate in the general data questionnaire survey, and sign an informed consent form.

### 2.3. Exclusion criteria

Patients with type 1 diabetes, gestational diabetes, or other specific types of diabetes.

Patients diagnosed with severe liver and kidney dysfunction, significant diseases such as cerebral hemorrhage, heart failure, or tumors, or those with severe physical illnesses, intellectual disabilities, mental disorders, or other conditions that prevent normal communication.

### 2.4. Data collection

A questionnaire survey and clinical indicator collection were conducted for the study participants. The general information survey form was designed by the researcher after reviewing extensive relevant literature and was finalized through discussions within the research team. The questionnaire consisted of 9 items: age, gender, marital status, place of residence, occupation, smoking history, drinking history, duration of diabetes, and family history. Clinical indicators were collected from the clinical medical record system of the Endocrinology Department’s inpatient ward, including physical indicators such as BMI and waist-to-hip ratio, as well as biochemical indicators like microalbuminuria, fasting plasma glucose, 2-hour postprandial blood glucose, glycated hemoglobin, triglycerides, total cholesterol, high-density lipoprotein cholesterol, low-density lipoprotein cholesterol, creatinine, and uric acid. Additionally, arterial ultrasound examination indicators, including Doppler ultrasound of both lower extremity arteries and veins and carotid artery Doppler ultrasound, were collected. Clinical complications were also recorded, with patients being clearly diagnosed with conditions such as diabetic nephropathy, diabetic retinopathy, coronary heart disease, and cerebral infarction.

### 2.5. Statistical methods

First, the dependent variable (whether hypertension is present) and independent variables (influencing factors) were assigned values. Univariate logistic regression analysis was then conducted for each independent variable, with a significance level of *P* < .05 used to select statistically significant variables. Variables found to be significant in the univariate analysis were further analyzed using multivariate logistic regression. The backward LR method was applied to identify the final variables to be included in the model and to construct the predictive model. As the primary goal of the analysis was to identify the model with the most significant predictive capability, the Hosmer–Lemeshow goodness-of-fit test in multivariate logistic regression was used to assess the model’s calibration, and a calibration curve was plotted. The predictive model was presented in the form of a nomogram. For internal validation of the predictive model, a bootstrap resampling method (with 1000 bootstrap resamples) was employed. The model’s performance was evaluated by measuring the Hosmer–Lemeshow goodness-of-fit test and calibration curve. The nomogram and calibration curve were generated using R programming language and environment version 4.0.2 (http://cran.r-project.org). Other statistical analyses were conducted using IBM SPSS Statistics for Social Sciences version 23.0. All statistical tests were two-sided, and *P* < .05 was considered statistically significant.

## 3. Results

### 3.1. Distribution of T2DM with hypertension

The 423 patients with T2DM were divided into 2 groups based on the presence of hypertension: the diabetes group and the diabetes with hypertension group. Among these, 206 patients (48.7%) were in the diabetes with hypertension group, and 217 patients (51.3%) were in the diabetes group. The details are shown in Table 1.

**Table 1:** Distribution of type 2 diabetes mellitus with hypertension.

Group	Cases	Proportion (%)
Type 2 diabetes with hypertension group	206	48.7
Type 2 diabetes group	217	51.3

### 3.2. Univariate logistic regression analysis of factors influencing T2DM with hypertension

Based on the results of the univariate analysis, the following variables were significantly associated with T2DM combined with hypertension: age, alcohol consumption, diabetic nephropathy, coronary heart disease, cerebral infarction, arteriosclerosis of the lower extremities, carotid atherosclerosis, BMI, waist-to-hip ratio, microalbumin, serum creatinine, and uric acid. The details are shown in Table 2.

**Table 2:** Logistic univariate regression analysis of type 2 diabetes mellitus with hypertension.

Factor	β	SE	Wald	*P*	OR	95% CI	
						Upper limit	Lower limit
Gender	0.165	0.198	0.834	.404	1.179	0.8	1.738
Marital status	0.352	0.323	1.088	.276	1.422	0.754	2.68
Occupation	-0.229	0.258	-0.887	.375	0.796	0.48	1.319
Residence	0.454	0.237	1.919	.055	1.575	0.99	2.505
Smoking	0.397	0.207	1.92	.055	1.487	0.992	2.23
Drinking	0.691	0.212	3.262	**.001**	1.996	1.318	3.024
Family history	-0.229	0.216	-1.06	.289	0.795	0.521	1.215
Diabetes duration	0.359	0.231	1.556	.12	1.432	0.911	2.252
Diabetic nephropathy	0.959	0.285	3.366	**.001**	2.609	1.493	4.559
Neuropathy	0.097	0.211	0.46	.646	1.102	0.728	1.667
Vascular complications	-0.167	0.195	-0.857	.391	0.846	0.577	1.24
Retinopathy	-0.123	0.302	-0.407	.684	0.884	0.489	1.599
Diabetic foot	1.171	0.822	1.424	.154	3.225	0.643	16.164
Coronary heart disease	1.632	0.432	3.777	**.001**	5.114	2.193	11.925
Hyperlipidemia	0.283	0.272	1.038	.299	1.327	0.778	2.263
Fatty liver	0.119	0.22	0.541	.588	1.127	0.731	1.736
Cerebral infarction	0.97	0.207	4.678	**.001**	2.637	1.757	3.958
Bilateral lower extremity arteriosclerosis	0.903	0.244	3.695	**.001**	2.467	1.528	3.984
Carotid arteriosclerosis	0.787	0.218	3.614	**.001**	2.197	1.434	3.366
Body mass index	0.753	0.199	3.785	**.001**	2.123	1.438	3.136
Waist to hip ratio	0.719	0.288	2.494	**.013**	2.052	1.166	3.611
Microalbumin	0.597	0.199	3.007	**.003**	1.817	1.231	2.682
Fasting blood glucose	0.223	0.253	0.881	.378	1.25	0.761	2.054
Postprandial 2 hour blood glucose	-0.056	0.391	-0.142	.887	0.946	0.439	2.036
Glycated hemoglobin	-0.057	0.349	-0.163	.87	0.945	0.477	1.871
Triglycerides	0.17	0.195	0.868	.385	1.185	0.808	1.737
Total cholesterol	-0.315	0.221	-1.427	.154	0.73	0.473	1.125
High density lipoprotein	-0.126	0.198	-0.637	.524	0.882	0.598	1.299
Low density lipoprotein	-0.318	0.39	-0.814	.415	0.728	0.339	1.564
Creatinine	1.373	0.375	3.659	**.001**	3.949	1.892	8.241
Uric acid	0.484	0.247	1.963	**.05**	1.623	1.001	2.632

The bold values represent factors that have a statistically significant association with the outcome (the coexistence of diabetes and hypertension). These factors have a *P*-value less than .05.

### 3.3. Multivariate logistic regression analysis of T2DM with hypertension

The multivariate logistic regression analysis identified the following factors as significant predictors of the risk of hypertension in patients with T2DM: age, alcohol consumption, diabetic nephropathy, coronary heart disease, cerebral infarction, and BMI. The details are shown in Table 3.

**Table 3:** Multivariate logistic regression analysis of type 2 diabetes with hypertension.

Factor	OR	SE	Wald	*P*	95% CI	
					Upper limit	Lower limit
(Intercept)	0.091	0.418	-5.751	**.001**	0.039	0.2
Age	1.887	0.26	2.439	**.015**	1.134	3.153
Drinking	1.78	0.241	2.394	**.017**	1.112	2.862
Diabetic nephropathy	2.131	0.361	2.097	**.036**	1.059	4.38
Coronary heart disease	4.619	0.471	3.251	**.001**	1.928	12.472
Cerebral infarction	1.729	0.245	2.231	**.026**	1.069	2.802
Bilateral lower extremity arteriosclerosis	1.299	0.317	0.824	.41	0.698	2.432
Carotid arteriosclerosis	1.56	0.289	1.539	.124	0.886	2.756
Body mass index	2.55	0.235	3.99	**.001**	1.618	4.066
Waist to hip ratio	1.441	0.333	1.096	.273	0.756	2.808
Microalbumin	1.206	0.25	0.749	.454	0.737	1.967
Creatinine	1.406	0.444	0.767	.443	0.602	3.478
Uric acid	1.462	0.293	1.296	.195	0.824	2.608

The bold values represent factors that have a statistically significant association with the outcome (the coexistence of diabetes and hypertension). These factors have a *P*-value less than .05.

### 3.4. Construction of the predictive model for hypertension in T2DM

**Figure 1.**
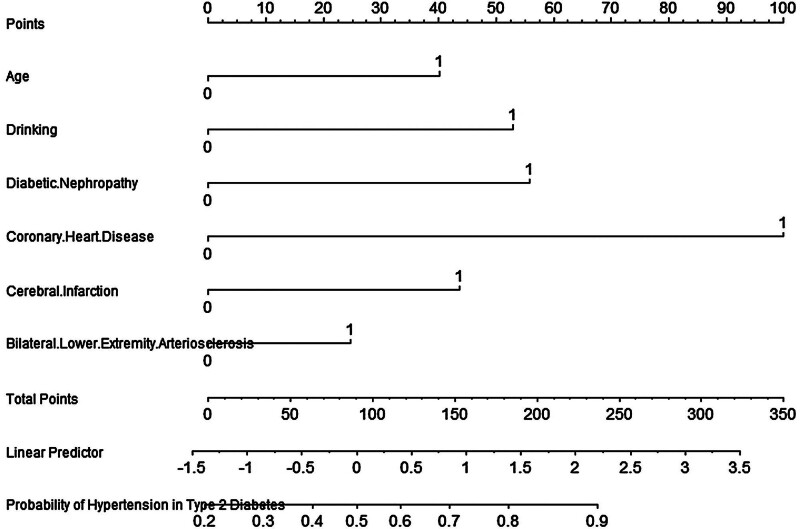
Nomogram for predicting the probability of hypertension in type 2 diabetes mellitus.

A predictive model was established using multivariate logistic regression. Out of the initial 32 variables, 6 variables were included in the predictive model as predictors: age, alcohol consumption, diabetic nephropathy, coronary heart disease, cerebral infarction, and BMI. The predictive model is presented in the form of a nomogram, which is used to estimate the probability of hypertension in patients with T2DM (Fig. 1).

The score calculation method is based on the regression coefficients from the multivariate logistic regression model. Each variable’s score is derived by converting the regression coefficient of that variable into a corresponding value on the nomogram. These regression coefficients reflect the relative contribution of each predictor to the risk of developing hypertension. For example, variables such as age, alcohol consumption, and the presence of coronary heart disease each have corresponding regression coefficients, which are mapped to specific score ranges. A patient’s specific characteristics (for example alcohol consumption or coronary heart disease) will have corresponding scores on the nomogram. The total score is the sum of all individual factor scores, and the probability is the estimated hypertension risk based on the total score.

The vertical axis represents the range of values for different predictors, while the horizontal axis shows the score corresponding to each value of the predictor, known as the “Score.” The total score indicates the sum of all predictor scores, and the probability represents the estimated hypertension risk based on the total score. For example, consider a 52-year-old patient with T2DM. The corresponding score for age is 0; for alcohol consumption, it is 53; for no diabetic nephropathy, it is 0; for having coronary heart disease, it is 100; for no cerebral infarction, it is 0; and for a BMI >24, it is 23. Summing these scores results in a total score of 176. According to the nomogram, this total score corresponds to a probability of approximately 78% for the patient to develop hypertension, indicating a relatively high likelihood of hypertension in this patient.

### 3.5. Performance validation of the predictive model for hypertension risk in T2DM

#### 3.5.1. Model discrimination validation

**Figure 2.**
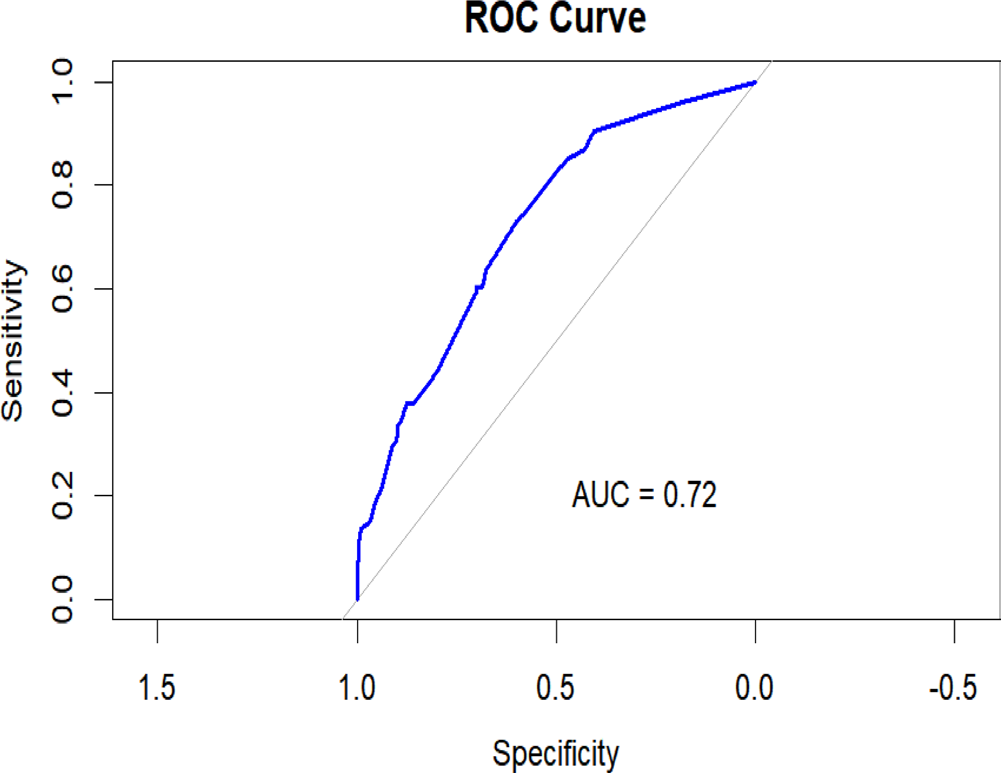
Calibration curve of the predictive model for hypertension risk in patients with type 2 diabetes mellitus.

The performance of the risk prediction model developed in this study was validated using ROC curves and other metrics. The ROC curve for the predictive model, which includes 6 risk factors: age, alcohol consumption, diabetic nephropathy, coronary heart disease, cerebral infarction, and BMI, shows an AUC of 0.72 (95% CI = 0.706–0.806, *P* < .001). This indicates that the predictive model constructed in this study has a good predictive ability (see Fig. 2).

#### 3.5.2. Model calibration validation

**Figure 3.**
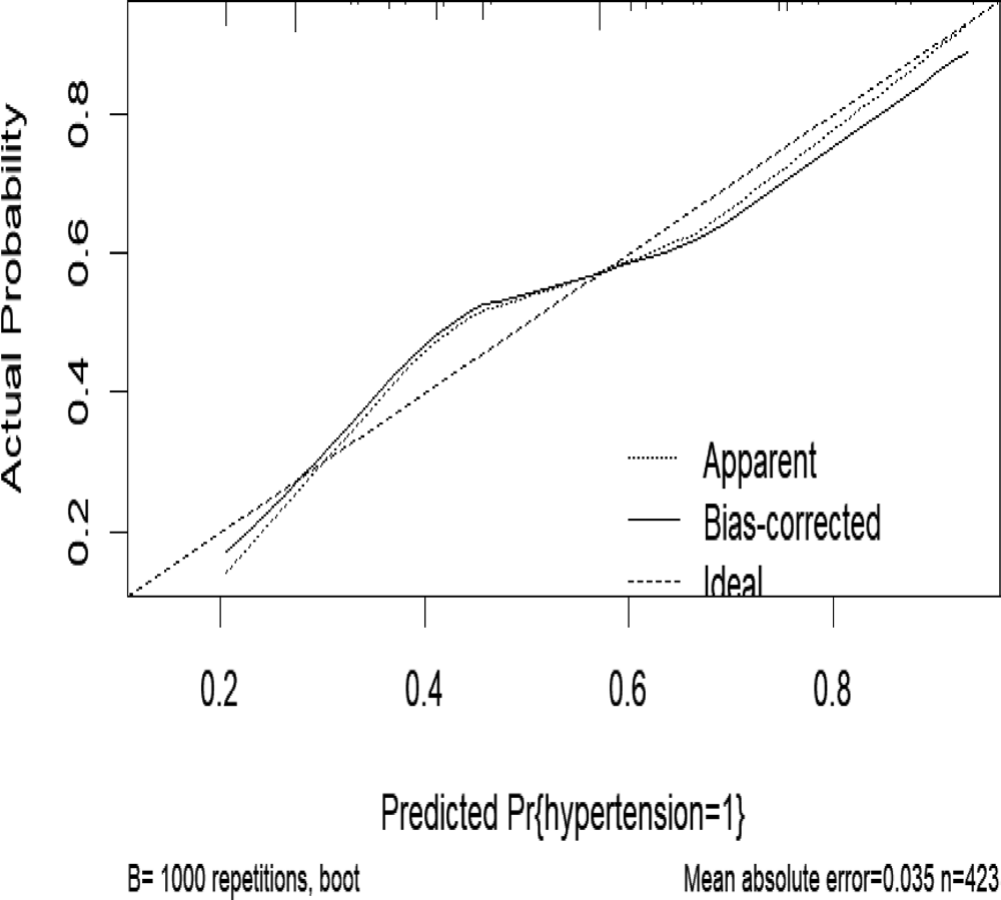
Calibration curve of the predictive model for hypertension risk in patients with type 2 diabetes mellitus.

Calibration is an essential indicator for evaluating the accuracy of a predictive model, reflecting the degree of consistency between the model’s predictions and actual observations. In logistic regression models, the Hosmer–Lemeshow test is commonly used to assess calibration. A more significant *P*-value from the test indicates better model fit, meaning the predicted probabilities align more closely with the actual observed probabilities. For the predictive model of hypertension risk in T2DM constructed in this study, the Hosmer–Lemeshow test resulted in χ² = 13.133 and *P* = .074. Since *P* > .05, there is no statistically significant difference between the predicted and observed values, suggesting that the model has good predictive performance. The calibration curve is shown in Figure 3.

## 4. Discussion

### 4.1. Prevalence of hypertension in T2DM

In this study, we found that the prevalence of hypertension in patients with T2DM was 48.7%, which is consistent with findings reported in other studies.^[7]^ Epidemiological research indicates that the prevalence of hypertension among T2DM patients typically ranges around 50%.^[8]^ Although there may be variations in prevalence across different regions and populations, the overall trend shows that a high comorbidity of hypertension among diabetes patients is common. Furthermore, some studies have reported that hypertension is 1.5 to 2 times more common in diabetes patients compared to nondiabetic individuals, which aligns with our findings.^[9]^ The prevalence of hypertension in our study is slightly lower than some reports indicating over 50%, which may be attributed to differences in region, sample size, and diagnostic criteria. Overall, these data underscore the importance of monitoring and controlling blood pressure in diabetes management, highlighting the widespread and high-risk nature of hypertension among diabetes patients.

### 4.2. Influencing factors of hypertension in T2DM

#### 4.2.1. Relationship between age and hypertension risk

This study demonstrates that age is a significant predictor of hypertension in patients with T2DM. This finding is consistent with numerous epidemiological studies indicating that older age is a considerable risk factor for hypertension.^[10]^ As individuals age, vascular elasticity decreases, vessel wall stiffness increases, and vascular resistance rises, leading to reduced arterial compliance.^[5]^ Additionally, older adults often experience metabolic dysfunction, elevated inflammatory markers, and endothelial dysfunction, all of which can further exacerbate the development of hypertension.^[11]^ A study on elderly populations found that the prevalence of hypertension significantly increases in those over 60 years of age and is associated with vascular stiffness, inflammatory markers, and insulin resistance.^[12]^ These findings suggest that special attention should be given to the prevention and management of hypertension in elderly patients with T2DM, including regular blood pressure monitoring, lifestyle modifications, and early pharmacological intervention.

#### 4.2.2. Impact of alcohol consumption on hypertension

Alcohol consumption has been established as a significant risk factor for hypertension, consistent with previous research findings.^[11]^ Numerous studies indicate a dose–response relationship between alcohol intake and elevated blood pressure, meaning that higher alcohol consumption is associated with higher blood pressure levels.^[11]^ This effect may be due to physiological changes caused by chronic alcohol use, such as excessive activation of the sympathetic nervous system, increased angiotensin II levels, and vasoconstriction, which contribute to elevated blood pressure.^[12]^ A cross-sectional study has shown that both systolic and diastolic blood pressures are significantly higher in moderate drinkers compared to nondrinkers.^[13]^ Based on this evidence, reducing or controlling alcohol intake should be a key component of hypertension prevention strategies for patients with T2DM, especially for those with existing hypertension or cardiovascular disease risk.

#### 4.2.3. Relationship between diabetic nephropathy and hypertension

Diabetic nephropathy is a common complication among patients with T2DM, and impaired kidney function can further increase the risk of hypertension.^[14]^ This study found that diabetic nephropathy is a significant predictor of hypertension, consistent with previous research.^[15]^ Patients with diabetic nephropathy are prone to elevated blood pressure due to decreased glomerular filtration rate, water and sodium retention, and activation of the renin–angiotensin system.^[15]^ Other studies have also shown that the incidence of hypertension is significantly higher in patients with diabetic nephropathy compared to those without renal complications.^[13]^ Therefore, early diagnosis and management of diabetic nephropathy, particularly the use of ACE inhibitors or ARBs to protect kidney function, are crucial for preventing the development of hypertension.

#### 4.2.4. Impact of coronary heart disease and cerebral infarction on hypertension

Coronary heart disease and cerebral infarction, as outcomes of atherosclerosis, are closely associated with hypertension.^[16]^ This study found that coronary heart disease and cerebral infarction significantly increase the risk of hypertension in patients with T2DM. Atherosclerosis leads to vascular stiffening and narrowing, which subsequently causes elevated blood pressure.^[17]^ Research shows that the prevalence of hypertension is significantly higher in patients with cardiovascular disease compared to those without, closely related to factors such as vascular wall thickening, endothelial dysfunction, and inflammation-mediated vasoconstriction.^[18]^ Therefore, more proactive blood pressure monitoring and management in individuals at high risk for coronary heart disease and cerebral infarction may be an essential strategy for preventing further cardiovascular events.

#### 4.2.5. Impact of BMI

This study confirms the significance of BMI in predicting the risk of hypertension in patients with T2DM. Obesity is not only a known risk factor for hypertension but is also associated with insulin resistance and chronic low-grade inflammation, which contribute to the development of hypertension.^[19]^ Increased fat tissue in obese individuals leads to activation of the sympathetic nervous system and excessive activation of the renin–angiotensin–aldosterone system, resulting in elevated blood pressure.^[20]^ A cohort study found a significant positive correlation between BMI and the risk of hypertension, with this risk being particularly pronounced in obese individuals.^[21]^ These findings suggest that clinical attention should be given to weight management in patients with T2DM, emphasizing lifestyle changes, increased physical activity, and dietary control to reduce the risk of hypertension.

### 4.3. Construction of the prediction model for hypertension in T2DM

The nomogram we developed provides a simple, easy-to-use, and personalized predictive model for hypertension in T2DM, aiding in the optimization of clinical management. This predictive score helps clinicians quickly assess the risk of hypertension based on a patient’s individual characteristics, thereby guiding treatment decisions. Through this model, clinicians can identify high-risk patients, enabling earlier and more proactive interventions to reduce the risk of disease complications.

Based on the 6 identified independent risk factors: age, alcohol consumption, diabetic nephropathy, coronary heart disease, cerebral infarction, and BMI, we have also proposed specific preventive and management measures. For older patients, regular blood pressure monitoring and health checkups are recommended, along with promoting a healthy diet and moderate exercise. For patients with alcohol consumption habits, it is advised to reduce or quit drinking, with psychological counseling support provided if necessary. In patients with diabetic nephropathy, strict blood glucose control is essential, and the use of renal-protective medications may be considered. For those with coronary heart disease, cardiovascular-protective drugs should be administered under medical guidance, accompanied by a low-salt, balanced diet and appropriate physical activity. Patients with a history of cerebral infarction should undergo comprehensive management of cardiovascular risk factors, with anticoagulant therapy used when necessary. For individuals with a high BMI, weight control through a low-calorie, high-fiber diet, and increased physical activity is recommended to reduce the risk of hypertension and related complications.

Simultaneously monitoring patients for the detection of hypertension allows for earlier identification of potential risks. By tracking changes in patients’ blood pressure, more timely and effective interventions can be implemented.

Internal validation using the bootstrap resampling method confirmed the model’s accuracy in predicting hypertension in T2DM (C-index = 0.757). Age, alcohol consumption, diabetic nephropathy, coronary heart disease, cerebral infarction, and BMI are independent risk factors for hypertension in T2DM. The model demonstrates strong predictive accuracy and discriminatory ability. However, although we have confirmed the reliability of the model through internal validation, it has not yet been validated in external populations, so further confirmation of its external validity may be needed.

## 5. Limitations

This study has several limitations in constructing the prediction model for hypertension in T2DM. Firstly, the sample size is relatively small and limited to a tertiary hospital in Anhui Province, which restricts the generalizability of the results. Additionally, as a cross-sectional study, it can only reveal correlations between variables and cannot establish causal relationships. Self-reported data may also introduce bias, potentially affecting the accuracy of the results. Some of the high-risk factors included in the study (such as age, diabetic nephropathy, coronary heart disease, and cerebral infarction) are non-modifiable and cannot be directly altered through interventions. However, these factors are still significant for identifying high-risk patients. Conversely, modifiable factors such as smoking and BMI can be improved through lifestyle interventions. Furthermore, the study did not account for temporal factors in disease progression, such as changes over time and the long-term effects of treatment. Despite these limitations, the study provides valuable insights into the prediction of hypertension risk in T2DM and lays the foundation for future research and model optimization.

## 6. Conclusion

This study constructed a predictive model for the risk of hypertension in T2DM based on regression analysis and validated its predictive ability and applicability. The results indicated that age, alcohol consumption, diabetic nephropathy, coronary heart disease, cerebral infarction, and BMI are independent risk factors for hypertension in T2DM patients. The nomogram model developed in this study provides a simple and practical tool for clinicians to rapidly identify high-risk patients, enabling early intervention and personalized management.

The findings not only confirm the high prevalence of hypertension in T2DM patients but also emphasize the importance of controlling modifiable factors, such as alcohol consumption and body weight. Additionally, the model’s excellent calibration and discrimination performance provide theoretical support for its application in clinical practice. Although this study has limitations in terms of sample size and regional scope, the results lay a foundation for future external validation studies involving larger, multicenter cohorts.

Future research should further optimize the model by incorporating longitudinal studies and new biomarkers to explore the dynamic mechanisms of hypertension in the progression of diabetes, providing more comprehensive support for the development of prevention and management strategies.

## Author contributions

**Writing – original draft:** Huiling Zhang.

**Data collection:** Dezheng Zhu.

**Resources:** Shuang Yu, Zheyuan Xia, Yahui Meng, Xiang Wang, Hui Shi.

**Software:** Huiling Zhang.

**Writing – review & editing:** Huiling Zhang.
